# Assessing the Hepatotoxic Effects of Fluoropyrimidine Chemotherapy in Male Iraqi Colorectal Cancer Patients

**DOI:** 10.7759/cureus.58126

**Published:** 2024-04-12

**Authors:** Muhtada A Challoob, Nawar S Mohammed

**Affiliations:** 1 Department of Clinical Biochemistry, University of Baghdad, College of Medicine, Baghdad, IRQ; 2 College of Pharmacy, University of Misan, Misan, IRQ; 3 Department of Biochemistry, University of Baghdad, College of Medicine, Baghdad, IRQ

**Keywords:** toxicity, hepatocyte, fluorouracil, crc, colorectal cancer

## Abstract

Introduction: Colorectal cancer (CRC) is one the most frequently occurring cancer types among various populations. Fluoropyrimidine is the backbone of first-line chemotherapy, the oral capecitabine, or intravenous 5-fluorouracil (5-FU) in various combinations and schedules the chemotherapy regime in the treatment of a wide variety of gastrointestinal cancers. The enzyme dihydropyrimidine dehydrogenase (DPD) functions as the rate-limiting step in the metabolism of fluoropyrimidine chemotherapies, and patients with complete or partial DPD deficiency are at increased risk of severe and fatal toxicity during treatment with fluorouracil.

Aim: This study aimed to examine the chemotoxicity of the 5-FU drug on hepatocytes in male Iraqi CRC patients.

Materials and methods: This research is a cross-sectional study conducted between November 2022 and April 2023. The study included 80 male participants who had undergone surgical intervention for stage III CRC under the care of the Misan Health Directorate, Misan Center for Tumors Treatment, located in Misan, Iraq. Based on their subsequent surgical treatment, the participants were divided into two groups. The first group, comprising 45 males aged between 41 and 71 years, experienced a relapse despite receiving adjuvant therapy, which involved a singular cycle of fluoropyrimidine-based chemotherapy (5-FU). The second group consisted of 35 male patients with CRC, aged between 40 and 57 years, who did not experience a relapse post-adjuvant therapy. Their adjuvant therapy involved a single round of fluoropyrimidine-based chemotherapy with 5-FU. Relapse in patients was determined by assessing the white blood cell count (WBC).

Results: Liver enzymes were significantly increased after 5-FU treatment, while the concentration of albumin was significantly decreased.

Conclusion: The findings of our study clearly indicate that 5-FU induced hepatic injury, lowering the hepatocyte function with elevated levels of hepatic enzymes and low concentration of albumin in the blood, which is an important predictive marker of chemotherapy toxicity.

## Introduction

Colorectal cancer (CRC) is one of the most frequently occurring cancer types among various populations. The incidence rate of CRC is steadily increasing each day. As a result, the estimated number of CRC survivors is also on the rise, projected to reach 2.5 million by 2035 [1]. In the past few decades, multiple factors, such as economic development, inappropriate lifestyle, and dietary habits, like consuming high amounts of red/processed meats, refined grains, fats, sugary foods, and vegetables, low amounts of dietary fiber, and fruits, resulting in the considerable increase in the CRC incidence [2]. Moreover, inflammation, physical inactivity, obesity, hence overweight, oxidative stress, smoking, and metabolic dysfunction are other factors affecting the incidence rate of CRC [3,4].

However, cancer cells grow and spontaneously proliferate faster than the immune system can handle, the cancer cells begin to build a microenvironment that starts at the time of cancer occurrence, and, in most cases, when a cancer mass is found, a tumor microenvironment is present, making it difficult for the immune system to efficiently eliminate cancer cells [5-7].

Fluoropyrimidine is the backbone of first-line chemotherapy, the oral capecitabine, or intravenous 5-fluorouracil (5-FU) in various combinations and schedules the chemotherapy regime in the treatment of a wide variety of gastrointestinal cancers, which provides higher response rates, longer progression-free survival, and better survival [8,9]. Potential toxicities associated with this type of chemotherapy include alopecia, mucositis, diarrhea, emesis, hand-foot syndrome, myelosuppression, and cardiac toxicity; these adverse reactions may be severe and fatal [10].

The liver is the major site of metabolism for many drugs, and this liver-drug interaction must be accounted for while dosing chemotherapy. Hepatocyte toxicity implies chemical-driven liver injuries [11]. Certain medicinal agents, when taken in overdoses and sometimes even when introduced within the therapeutic ranges, may injure the organ. The hepatocyte participates in a variety of metabolic activities and contains a host of enzymes [12]. The liver enzymes, including alanine aminotransferase activity (ALT), aspartate aminotransferase activity (AST), and alkaline phosphatase activity (ALP), have been shown to be a biomarker of hepatic injury [13].

In this research, we investigated the hepatotoxic effects of the drug 5-FU on hepatocytes in male patients with CRC in Iraq.

## Materials and methods

Study design and participants

This study is a cross-sectional study, enrolling participants who were patients under the care of the Misan Health Directorate, Misan Center for Tumors Treatment, located in Misan, Iraq. The research was carried out from November 2022 to April 2023, encompassing a group of 80 male participants from Iraq who had previously undergone surgical intervention for stage III CRC according to their subsequent surgical treatment. They were divided into two distinct groups: The first group consisted of 45 males, aged between 41 and 71 years. These participants encountered a relapse despite receiving adjuvant therapy in the form of a singular cycle of fluoropyrimidine-based chemotherapy (5-FU). The second group comprised 35 male patients with CRC. Their ages ranged from 40 to 57 years, and these participants had not encountered relapse post-adjuvant therapy. The therapy encompassed a solitary round of fluoropyrimidine-based chemotherapy involving 5-FU. Patient categorization in cases of relapse was established through the assessment of white blood cell (WBC) counts. The participants received detailed information about the research goals, and data compilation occurred via an extensive questionnaire. Notably, the study strictly adhered to ethical protocols endorsed by the Ethics Committee of the College of Medicine at the University of Baghdad. The participants were provided with comprehensive details regarding the research objectives, and data collection was carried out through an in-depth questionnaire. Importantly, the study rigorously followed ethical guidelines approved by the Ethics Committee of the College of Medicine at the University of Baghdad (IRB No. 2023/136A).

Exclusion criteria

All the individuals (patients) who had kidney cancer, bladder cancer, lung cancer, prostate cancer, pancreatic cancer, stomach cancer, melanoma, thyroid cancer, leukemia, brain cancer, liver cancer, bone cancer, liver disease, or active inflammatory conditions were excluded. Moreover, patients with Cushing's disease, chronic pancreatitis, acromegaly, chronic renal failure, pancreatectomy, and chronic or acute inflammatory disease and patients who were taking drugs, had a history of smoking, underwent lipid-lowering therapy, or were drinking alcohol were excluded.

Blood sample collection and processing

Vein blood of 5 ml was collected from the elbow of each subject using plastic disposable syringes in the morning. Blood samples were collected in gel tubes with an activator for collating blood and allowed to clot at room temperature for approximately four minutes. The serum was separated by centrifugation (4000 rpm) for 10 minutes, and then the serum was isolated within an hour of blood collection, which was then immediately transferred to another tube (divided into three small Eppendorf tubes) and stored at -20°C for subsequent analyses. The hemolyzed samples were excluded, and before analyses, the samples were allowed to attain room temperature. The body mass index (BMI) was estimated in kg/m^2^ and was calculated using height and weight data by dividing the person's weight in kilograms by the height in meters squared. Everyone was weighed barefoot on the same analog weight scale.

Biochemical analyses

The alanine transaminase (ALT), aspartate aminotransferase (AST), and alkaline phosphatase (ALP) in the serum were estimated according to the method described by Murray and Kaplan; the ALP activity in the serum was estimated according to the method described by Wenger; the albumin concentration in serum was estimated according to the method described by Doumas. All these methods used kits manufactured by Spinreact, Ctra. Sta. Coloma, Spain. The WBC counts were estimated by an automated hematology analyzer.

Statistical analyses

The collected data were analyzed using the IBM SPSS Statistics for Windows, version 23.0 (released 2015, IBM Corp., Armonk, NY). Descriptive data including mean, standard deviation, and the correlation between the two groups were calculated. The correlation coefficient (r) among the parameters was calculated, and a p-value ≤ 0.05 was considered statistically significant.

## Results

The study participants’ ages ranged between 41 and 71 years old for relapsed patients and 40 and 57 years old for non-relapsed patients. The results presented in Table 1 show that there are no significant differences in these parameters (ages and BMI) between the relapsed patients and non-relapsed patients (p > 0.05). Moreover, the results demonstrate the presence of a highly significant decrease in WBCs in the relapsed patients in comparison with that of the non-relapsed patients (p < 0.001).

**Table 1:** Difference between the characteristics of relapsed patients and non-relapsed patients BMI: body mass index, WBC: white blood cell. ** Statistically highly significant

Individual characteristics	Relapsed patients (N = 45)	Non-relapsed patients (N = 35)	p-value
	Mean ± SD	Mean ± SD	
Age (years)	56.13 ± 9.03	48.00 ± 5.12	0.061
BMI (kg/m^2^)	20.40 ± 2.82	19.29 ± 2.33	0.052
WBC (10^9^/L)	2.247 ± 0.253	3.842 ± 2.251	0.001**

**Figure 1:**
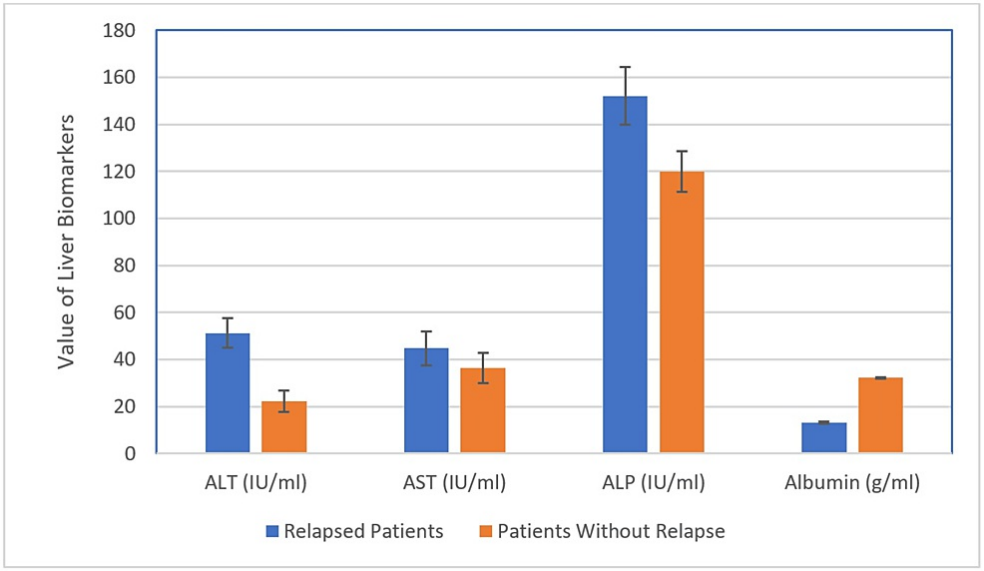
Variation in liver biomarkers between the relapsed and non-relapsed patients. ALT: alanine aminotransferase, AST: aspartate aminotransferase, ALP: alkaline phosphatase, DPD: dihydropyrimidine dehydrogenase

The results in Table 1 demonstrate the presence of a highly significant increase in serum ALT and highly significant decrease in serum albumin of the relapsed patients in comparison with that of the non-relapsed patients (p < 0.001). These results also demonstrate that there is a significant increase in AST and ALP in the serum of the relapsed patients compared with that of the non-relapsed patients (p < 0.05). These results are also illustrated in Figure 1.

## Discussion

The present study showed significant hepatocyte damage elicited by the increased level of serum marker enzymes and aminotransferase enzymes in patients by treatment with capecitabine alone in comparison with healthy males. The elevated serum levels of the hepatic biomarkers have been attributed to liver damage because these enzymes are released into circulation in the case of hepatocellular damage [13].

Albumin concentration was a significant statistical predictor for hepatotoxicity because only the liver is the site of albumin synthesis; therefore, when the hepatic cells are damaged, they cannot synthesize albumin normally. Decreased albumin concentration is correlated with higher toxicity of chemotherapy and poor survival [14].

The results of serum albumin are in agreement with the study done by Srdic et al., who studied the albumin concentration in serum patients with lung cancer after treatment with chemotherapy [15]. While these results disagree with the study by Zhu et al., there was no significant change in the albumin level in patients with locally advanced rectal cancer who received capecitabine treatment [15].

The liver is the major source of albumin synthesis. A decrease in serum albumin possibly suggests inflammation, chronic infections, kidney problems, and cirrhosis, among others [16,17]. The decreased albumin concentration in the patients suggests that some level of hepatocyte damage may occur following chemotherapy exposure. Normally, the lowering of albumin level indicates a liver disease [16]. This reduction could be attributed to changes in the free amino acid and protein metabolism and their synthesis in the liver. The lowering of protein may be due to dysfunction in the mechanism synthesis of hepatocyte protein and the hyperactivity of hydrolytic enzymes [16,17]. The decreased levels of albumin suggested that fluorouracil may induce hepatotoxicity and nephrotoxicity by interfering metabolic activities and synthesis of protein [16,17].

Experimental application of fluorouracil can induce DNA damage and apoptosis in the cell, causing excessive free radicals [18]. Many studies have reported that oxidative stress plays a very important role in fluorouracil induced to toxicity and renal toxicity [18-20]; therefore, loss of albumin through the renal and depletion it by reactive oxygen species.

A lower concentration of albumin observed following administration of fluorouracil is indicative of the consequences of lower protein synthesis via hepatocyte dysfunction or increased protein loss through the gastrointestinal or renal tract [21].

Less than 60% of capecitabine drug and its metabolites are attached to plasma proteins, and 35% are bound to albumin. This binding is independent of drug levels in the blood [21]. As expected, being a pro-drug of fluorouracil, capecitabine is also metabolized by the dihydropyrimidine dehydrogenase (DPD), which converts 5-FU to the much less toxic form known as 5-fluoro-5,6-dihydro-fluorouracil to 5-fluoro-ureido-propionic acid and finally the ultimate catabolites mainly α-fluoro-β-alanine; therefore, any genetic defect in this enzyme leads to hepatotoxicity because of accumulating these drugs [22-24]. Fluorouracil is associated with mitochondrial membrane collapse and a reduction in membrane potential that might impair the oxidation of fatty acids and lead to subsequent accumulation of reactive oxygen species (ROS) within hepatocytes [25]. 5-FU is also associated with the generation of ROS by microsomal cytochrome P450 enzymes. Furthermore, 5-FU is metabolized into catabolites, such as fluoro-β-alanine, which might decrease the capacity of hepatic cells to metabolize substances, such as drugs and lipids [26]. The mechanism of chemotherapy-induced hepatocyte damage is thought to be secondary to the production of ROS, intended to induce tumor cell apoptosis [27].

The increased activity of plasma AST, ALT, and ALP levels in the 5-FU-treated patients is a manifestation of induced hepatocyte damage. The higher levels of ALP activity are generally associated with the impairment of intrahepatic or hepatobiliary injury because of the prolonged destruction of hepatic cells [17].

Our findings of ALT, AST, and ALP levels agree with some studies, such as a study done by Vincenzi et al., who studied the serum ALT, AST, and ALP levels in colorectal cancer with hepatotoxicity by the folinic acid, fluorouracil, and irinotecan (FOLFIRI) treatment [26]. Moreover, our results are in agreement with the study of Khalid et al., who studied the dosage adjustments for chemotherapy in colorectal and pancreatic cancer patients [28].

Limitations

This study has limitations, notably being a single-center investigation, leading to a relatively small sample size.

## Conclusions

The conclusive results of this study unequivocally demonstrate that 5-FU induces hepatic injury, manifested in heightened enzyme activities in patients' bloodstream and reduced concentrations of albumin, an essential predictive marker of chemotherapy toxicity. The application of this method in clinical settings holds promise for informing the selection of appropriate oncologic treatments. As a result, dose adjustments may be necessary, including potential drug dose reduction or cessation of treatment. This decision should be based on regular monitoring of liver function tests both before initiating fluoropyrimidine-based chemotherapy and throughout the treatment period to prevent patient relapse.
